# Supplementary material for: Limosilactobacillus reuteri Attenuates Atopic Dermatitis via Changes in Gut Bacteria and Indole Derivatives from Tryptophan Metabolism
Source: Int J Mol Sci. 2022 Jul 13;23(14):7735. doi: 10.3390/ijms23147735 (PMC9320942; doi:10.3390/ijms23147735)
Supplement: Supplementary file 1 [file ijms-23-07735-s001.zip › ijms-1814813-supplementary.pdf]

Supplementary Material

1 Supplementary Figure

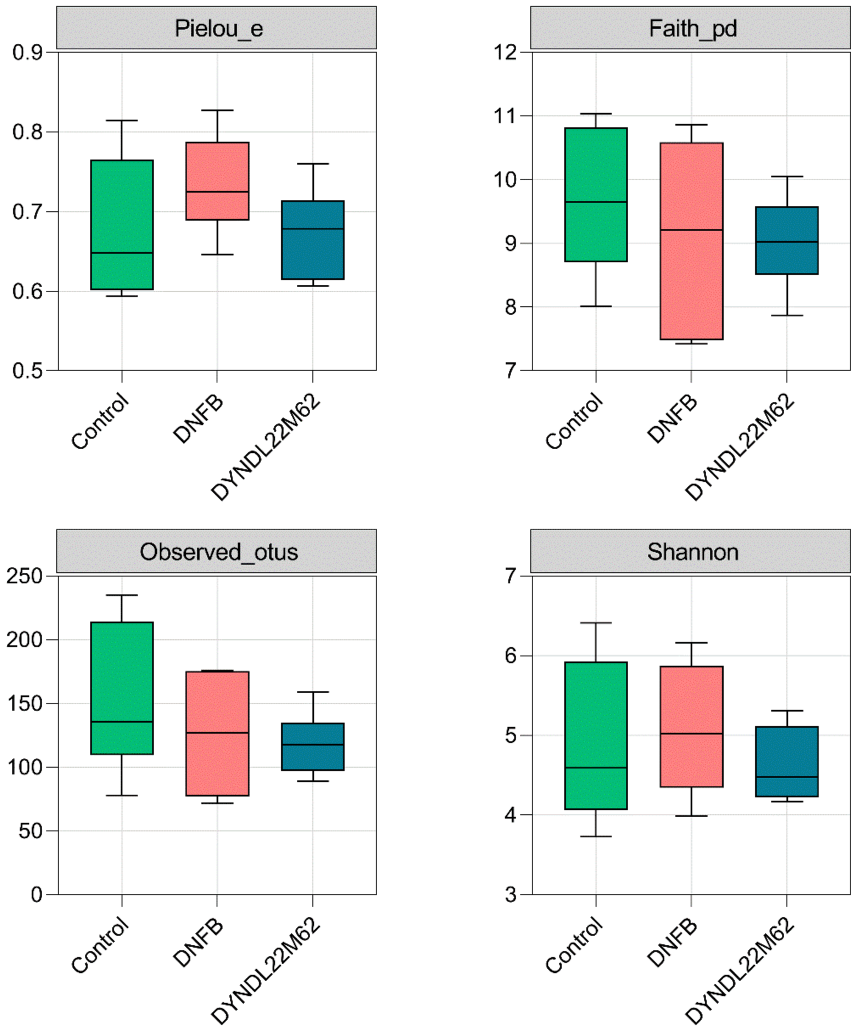

Supplementary Figure S1. Alpha diversity of gut microbiota between groups.
